# Supplementary figures and images for: Proinflammatory Endothelial Phenotype in Very Preterm Infants: A Pilot Study
Source: Biomedicines. 2022 May 20;10(5):1185. doi: 10.3390/biomedicines10051185 (PMC9138391; doi:10.3390/biomedicines10051185)

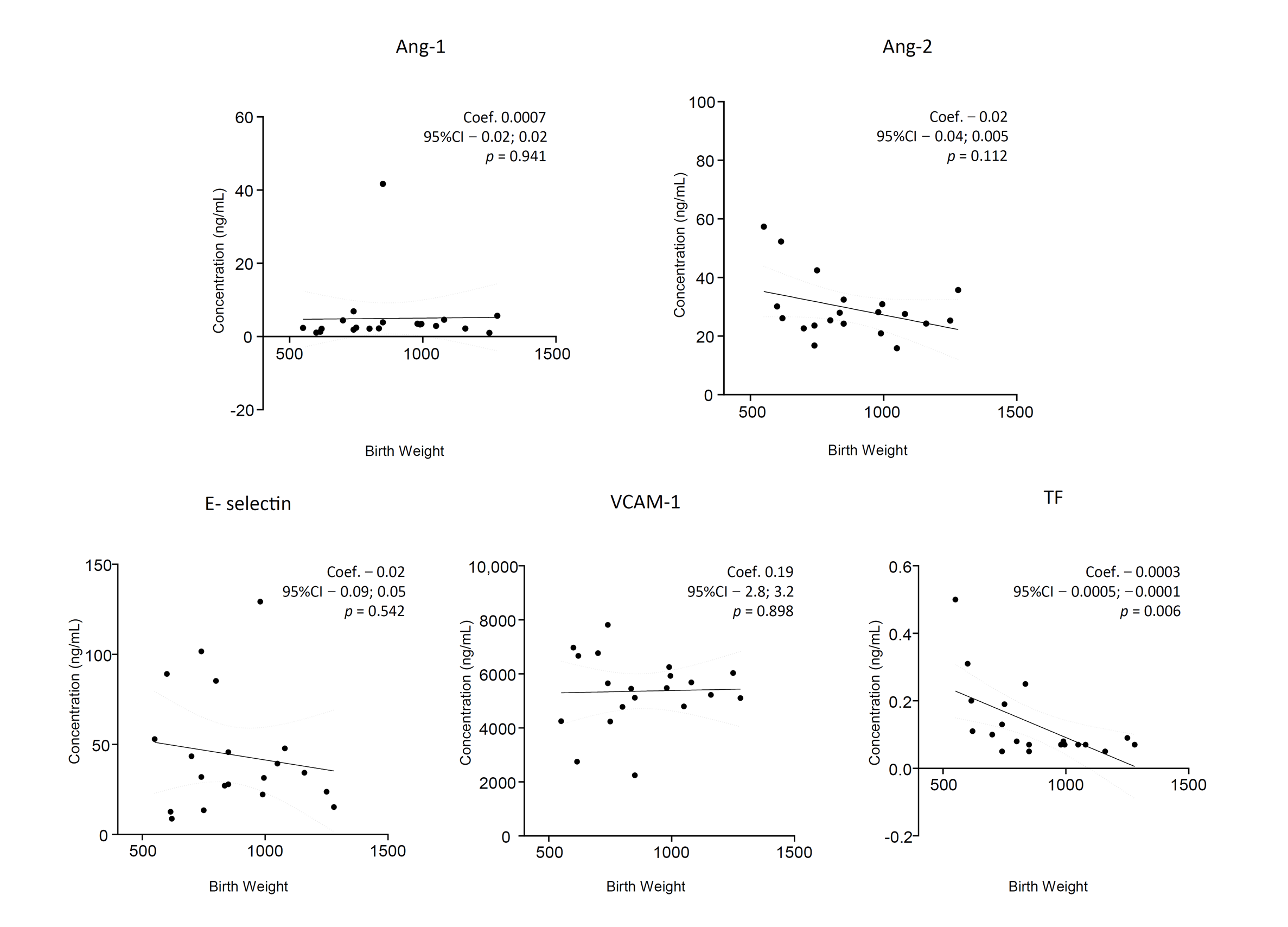

Supplement: Supplementary file 1 [file biomedicines-10-01185-s001.zip › biomedicines-1725800-supplementary.tif]
